# Thermo-hydraulic performance optimization of a disk-shaped microchannel heat sink applying computational fluid dynamics, artificial neural network, and response surface methodology

**DOI:** 10.1016/j.heliyon.2023.e21031

**Published:** 2023-10-17

**Authors:** Kourosh Vaferi, Mohammad Vajdi, Sahar Nekahi, Amir Heydari, Farhad Sadegh Moghanlou, Hossein Nami, Haleh Jafarzadeh

**Affiliations:** a Department of Mechanical Engineering, University of Mohaghegh Ardabili, Ardabil, Iran; b Department of Chemical Engineering, University of Mohaghegh Ardabili, Ardabil, Iran; c SDU Life Cycle Engineering, Department of Green Technology, University of Southern Denmark, Campusvej 55, 5230 Odense M, Denmark; d Department of Civil Engineering, School of Science and Engineering, Khazar University, Baku 1096, Azerbaijan

**Keywords:** Bionic fractal heat sink, Thermal and hydrodynamic properties, Response surface methodology, Artificial neural network, Python

## Abstract

The current research focuses on optimizing the Nusselt number (*Nu*) and pressure drop (*ΔP*) in a bionic fractal heat sink. The artificial neural network (ANN) and response surface methodology (RSM) were used to model the thermos-hydraulic behavior of the MCHS. The aspect ratios of *t/b* (cavities' upper side to bottom side ratio) and *h/b* (cavities’ height to bottom side ratio), as well as the Reynolds number, were set as the independent variables in both ANN and RSM models. After finding the optimum state for the copper-made MCHS (containing the optimum design of the cavities along with the best applied velocity), different materials were tested and compared with the base case (heat sink made of copper). The obtained results indicated that both ANN and RSM models (with determination coefficient of 99.9 %) could exactly anticipate heat transfer and *ΔP* to a large extent. To achieve the optimal design of the microchannel heat sink (MCHS) with the objective of maximizing *Nu* and minimizing *ΔP*, the efficiency index of the device was evaluated. The analysis revealed that the highest efficiency index (1.070 by RSM and 1.067 by ANN methods) was attained when the aspect ratios were *t/b* = 0.2, *h/b* = 0.2, and the Reynolds number was 1000. Next, the effect of the different materials on heat sink performance was investigated, and it was observed that by reducing the thermal conductivity, the thermal resistance of the heat sink increased and its overall performance decreased.

## 1 Introduction

Overheating has a significant risk to the functionality and durability of advanced microelectronic devices and chips. Any form of excessive temperature increase can lead to undesired distortion or even complete malfunctioning, ultimately compromising their reliability and overall lifespan [[1], [2], [3]]. The ambient temperature, the heat removal efficiency of the device, and the rate of heat generation of electronic components are the main reasons for device temperature [4,5]. Controlling the electronic devices’ temperature will ensure that they are kept cool and give trouble-free operation. Microchannel heat sinks (MCHSs) are innovative techniques to increase the heat removal rate to keep components within permissible operating temperature limits and enhance their longevity [[6], [7], [8]]. Smaller channel size in the MCHSs provides a higher surface-area-to-volume ratio and results in a considerable heat dissipation rate [9,10].

The application of single-layered MCHSs for heat elimination was firstly investigated by Tuckerman and Pease [11], and ever since, various MCHS designs have been innovated to meet the cooling demand for microelectronic equipment [12,13]. Different microchannel geometries have been utilized to enlarge the heat transfer area. Various materials with excellent thermal conductivity have been applied to make the MCHSs [14]. Different types of coolant with higher heat removal capabilities than air have been tested to enhance the cooling capacity [[15], [16], [17]]. The coupling effects of different nanofluids and substrate materials on thermo-hydraulic performance in an MCHS with trapezoidal channels were examined by Mohammed et al. [18]. They perceived that using glycerin-based nanofluids in a steel-made heat sink could exhibit more temperature uniformity and a more significant heat transfer coefficient. In another study, they analyzed the role of applying different nanofluids on the heat transfer capacity of an MCHS with triangular channels [19]. According to their results, the utilization of diamond nanoparticles within the water decreased the maximum temperature and improved the overall heat transfer performance in aluminum-made MCHS.

Bionic fractal heat sinks have gained attention from researchers due to their uniform velocity distribution and enhanced heat dissipation abilities. These characteristics make them a favorable choice among various designs of MCHS [20]. These types of MCHSs were first introduced by Adrian Bejan and investigated frequently in recent years [21]. The *ΔP*, heat transfer, and thermal resistance in four various types of MCHSs were studied by Xu et al. [22]. They noticed that the tree-like MCHS significantly ameliorated the heat transfer features and decreased the *ΔP*. Besides changing the geometry of the channel, heat transfer augmenters such as fins, reentrant cavities, and ribs are utilized to enhance heat transfer. Nevertheless, this increment comes at the cost of a more significant *ΔP* and pumping power demands. The roll of several fan-shaped ribs on the thermal and hydrodynamic performances of an MCHS were examined by Chai et al. [23]. They realized that the height and spacing of the fan-shaped ribs remarkably affected the temperature distribution and heat transfer coefficient. Furthermore, the utilization of the mentioned ribs on the walls of the MCHS could elevate the average *Nu* by about 101 %. Heat transfer and *ΔP* behavior in a micro heat exchanger with fan-shaped cavities were experimentally studied by Pan et al. [24]. They found out that the deviation degree, distribution of cavities, and their coincidence degrees greatly influenced the thermal efficiency of the device. The simultaneous impacts of the bionic fractal and several cross-sections on heat transfer and flow properties of the tree-shaped MCHS with ribs and cavities were examined by Huang et al. [25]. The authors pointed out that although the application of ribs on the sidewalls of the tree-shaped MCHS could increase the heat transfer performance, the higher *ΔP* was occurred. However, using cavities on the microchannels of the tree-shaped MCHS resulted in the best overall performance of the device.

Conducting parametric experimental study on MCHSs, needs fabrication of several MCHSs, which is costly, and time consuming. In addition, it is difficult to obtain detailed data at each point of the MCHS. However, numerical studies provide a way to solve problems quickly and easily compared to experimental ones [26,27]. Over the years, researchers have been examined and proposed different approaches to develop numerical schemes to accelerate the solution of complex equations or optimize the behavior of variables. The finite element method is a common approach utilized in computational fluid dynamic (CFD) codes to speed the solution [28,29]. The goal of these numerical schemes is the design of techniques to predict approximate but accurate solutions. Recently, there has been a growing interest in utilizing two different models, namely artificial neural network (ANN) and response surface methodology (RSM), to optimize the numerical or experimental responses [30]. The ANN that has stepped into the world in the mid-20th, is a computational scheme utilized to develop algorithms for modeling complex patterns [31]. The other effective technique to optimize the parameters and specify the best operating condition in the engineering processes is RSM [[32], [33], [34]] This scheme allows assessing the impacts of multiple parameters and their interactions on the examined responses [[35], [36], [37]].

The thermo-hydraulic features in a shell-and-tube heat exchanger with wavy tapes and plate baffles were studied by Yu et al. [38]. The width, pitch, and amplitude of tapes and the Reynolds number were the four variables of the RSM models investigated in this study. According to 25 CFD runs determined by central composite design (CCD) of RSM, the wavy tapes increased heat transfer coefficient via producing vortexes. The optimum shape of the inner corrugated tubes in a double-pipe heat exchanger were examined by Han et al. [39]. They used RSM method and observed that the optimum design of tubes was acquired for corrugation pitch of 0.82, corrugation height of 0.22, and corrugation radius of 0.23 at the Reynolds number of 26263. The thermal performance and *ΔP* behavior in a triple concentric-tube heat exchanger were accurately anticipated by Moya-Rico et al. [40]. The back-propagation algorithm of ANN models was selected to be utilized in the network's training, and the depth and pitch of corrugated tubes were the investigated variables. The authors understood an acceptable accord between the ANN data and experimental outcomes with the absolute average relative deviation of 1.91 % and 3.82 % for the heat transfer coefficient and *ΔP*, respectively.

Despite extensive research in MCHSs, the heat transfer enhancement in these devices is still of high importance. In the current study, heat transfer enhancement in a bionic fractal MCHS is investigated. To optimize the thermos-hydraulic performance, two ANN models with a 3-6-1 architecture and two quadratic models of RSM are employed to anticipate the Nusselt number and pressure drop. These prediction models are also used to find the optimum design of trapezoidal cavities and the best-applied velocity. Utilizing the mentioned ANN and RSM models as a predictive tool, the current work offers researchers an efficient and cost-effective means of accelerating their investigations in the related fields. After finding the optimum state for the copper-made MCHS, different materials are tested and compared with the base case (heat sink made of copper).

## 2 Methodology

### 2.1 Structural characteristics of the MCHS

**Fig. 1:**
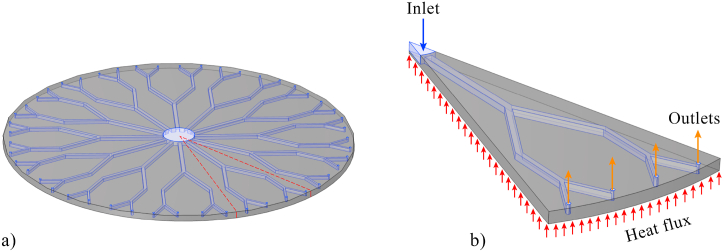
Structure of (a) the disk-shaped MCHS and (b) the simulated model.

In this study, a disk-shaped heat sink with tree-like microchannels which falls under the category of a specific type of fractal bionic MCHSs is analyzed. The design of the MCHS is adopted from the investigation conducted by Huang et al. [25]. Fig. 1a illustrates the schematics of the analyzed heat sink. It comprises a top cover, a substrate with microchannels arranged like a tree, and a heat source positioned beneath this substrate. Trapezoidal cavities are utilized on the side walls of the microchannels to enhance the heat transfer characteristics of the device. Due to the symmetrical design of the disk-shaped MCHS, only a one-twelfth of the entire heat sink is analyzed, as shown in Fig. 1b. The simulated model consists of an entry point, four exit points, interconnected microchannels, and trapezoidal cavities present on the side walls of these microchannels. *t/b* indicates the cavities' upper side to bottom side ratio, while *h/b* shows cavities’ height to bottom side ratio. *L*_*0*_ refers to the length of the microchannels in level 0 of the device, and the lengths in subsequent levels are determined using the following equation:

(1) $$\beta =\frac{L_{i+1}}{L_{i}}$$

**Fig. 2:**
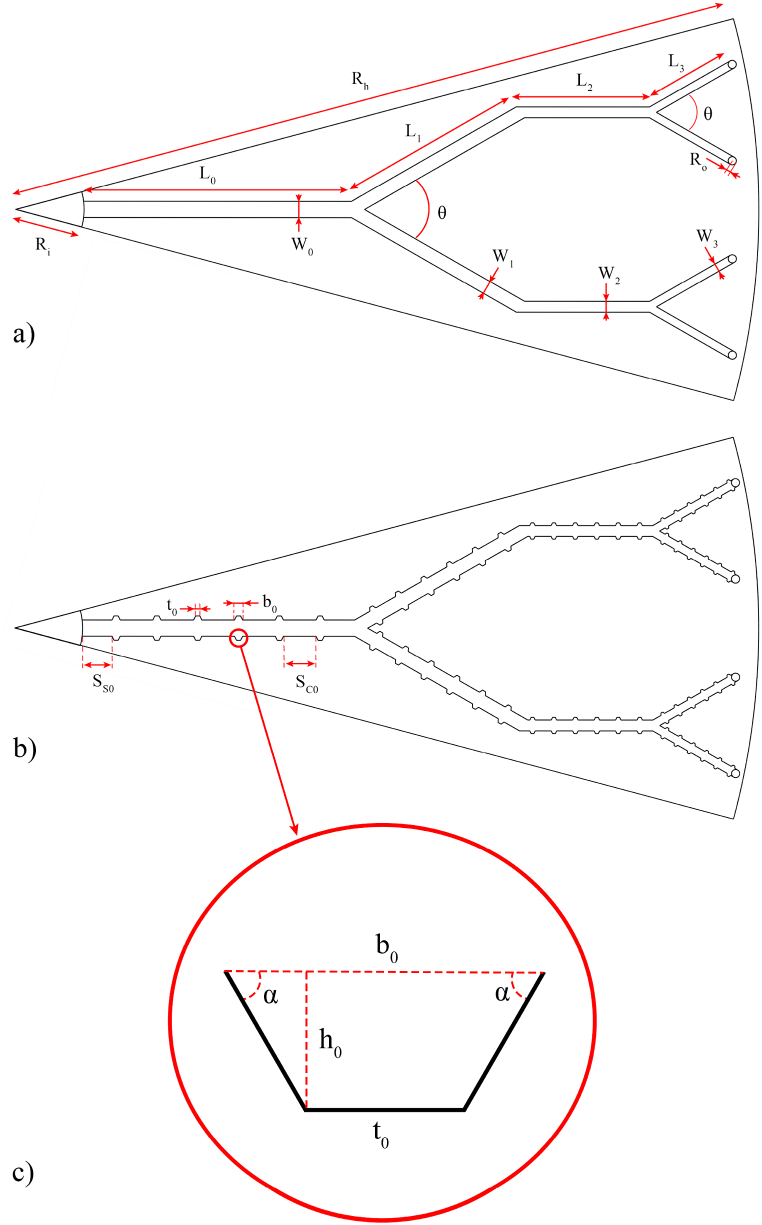
The geometrical parameters of (a) the simple microchannels, (b) the microchannels with cavities, and (c) the trapezoidal cavity.

Fig. 2a–c illustrate the geometric characteristics of the tree-like microchannels and the trapezoidal cavities. In detail, the dimensions of these parameters are presented in Table 1. Based on the information provided in Table 1, *H*_*h*_ corresponds to the height of the heat sink, while *H*_*c*_ represents the height of the microchannels. *R*_*h*_ represents the radius of the device, whereas *R*_*i*_ and *R*_*o*_ refer to the radiuses of the inlet and outlet of the microchannels, respectively. The trapezoidal cavities are evenly positioned with a distance of *S* between each other. The present numerical simulation utilizes the following scaling relations to define the parameters of subsequent levels. The subscript *i* shows the number of microchannel's level in the disk-shaped MCHS. In the presented relations, *b* and *W* denote the length of the cavities' bottom side and the width of microchannels, respectively.

(2) $$\beta^{\prime }=W_{i}-W_{i+1}$$

(3) $$\beta =\frac{S_{C_{i+1}}}{S_{C_{i}}}$$

(4) $$\beta^{\prime \prime }=\frac{S_{S_{i+1}}}{S_{S_{i}}}$$

(5) $$b_{i}=\frac{W_{i}}{2}$$

**Table 1:** Dimensions of the simulated parameters.

Parameter	Value	Parameter	Value
*R*_*h*_ (mm)	27.5	*β*	0.7
*R*_*i*_ (mm)	2.5	*β′*	0.1
*R*_*o*_ (mm)	0.15	*β’’*	0.6
*H*_*c*_ (mm)	0.5	*θ*	60^◦^
*H*_*h*_ (mm)	0.9	*α*	45^◦^
*L*_*0*_ (mm)	10	*S*_*C0*_ (mm)	1.2
*W*_*0*_ (mm)	0.6	*S*_*S0*_ (mm)	1.1

### 2.2 The governing equations

The tree-like microchannels in the disk-shaped heat sink are exposed to a high amount of heat fluxes from the disk's bottom surface. The heat is transferred into the microchannels from the heat source and is then dissipated by the coolant which flows within these microchannels. In the current numerical research, Computational Fluid Dynamic (CFD) is used to examine the flow pattern and temperature profiles in the disk-shaped MCHS. The conservation equations for fluid and solid are presented by Eqs. (6)–(9) [41]:

(6) $$\rho_{f}\nabla .\vec{U}=0$$

(7) $$\rho_{f}\vec{U}.\nabla \vec{U}=-\nabla P+\nabla .\left(\mu \nabla \vec{U}\right)$$

(8) $$\rho_{f}c_{p}\vec{U}.\nabla T=\nabla .\left(k_{f}\nabla T\right)$$

(9) $$k_{S}\nabla^{2}T=0$$

In the above-mentioned equations, *U*, *P*, and *T* represent the velocity, pressure, and temperature of the coolant, respectively. The density (*ρ*_*f*_), dynamic viscosity (*μ*), heat capacity (*c*_*p*_), and thermal conductivity (*k*_*f*_) as the thermo-physical properties of the water (coolant) are considered to be temperature-dependent [25]. The disk-shaped MCHS is made of copper and *k*_*s*_ represents the thermal conductivity of the copper. The constant thermo-physical features of the materials which have been investigated in the current study are given in Table 2.

**Table 2:** The thermo-physical features of the investigated materials at 298.15 K.

Material	*ρ* (kg/m^3^)	*C*_*p*_ (J/kg.K)	*k* (W/m.K)
Copper [25]	8940	380	387.6
Aluminum [42]	2719	871	202.4
Silicon [12]	2329	702	148
TiB_2_ [2]	4520	636	94.3
ZrB_2_ [3]	6080	429	83.5
Alumina [43]	3850	765	35

To facilitate solving Eqs. (6)–(9), several basic assumptions are taken into account.

• Laminar, steady, viscous, incompressible, and Newtonian flow.
• No temperature jump and non-slip condition at sidewalls.
• Uniform heat flux of 10^5^ W/m^2^ at the basal wall.
• The constant temperature of 298.15 K at the inlet.
• Adiabatic condition of the other walls.

The Reynolds number (*Re*) of a flowing fluid is a criterion to examine the flow regime and is defined by Eq. (10).

(10) $$Re=\frac{\rho_{f}{UD}_{i}}{\mu }$$

where *D*_*i*_ is the inlet diameter of the MCHS. The value of hydraulic diameter for the level *i* of microchannels and its average value can be calculated by Eqs. (11), (12), respectively.

(11) $$D_{hi}=\frac{2W_{i}H_{c}}{W_{i}+H_{c}}$$

(12) $${\bar{D}}_{h}=\frac{D_{0}L_{0}+2D_{1}L_{1}+2D_{2}L_{2}+4D_{3}L_{3}}{L_{0}+2L_{1}+2L_{2}+4L_{3}}$$

*Nu* and heat transfer coefficient (*HC*) as parameters to exhibit the heat transfer rate are formulated by Eqs. (13)–(15). The total thermal resistance (*R*_*t*_) is considered as a remarkable criterion used to assess the effectiveness of heat transfer in the MCHSs and expressed as follows:

(13) $$Q=\dot{m}c_{p}\left(T_{out}-T_{in}\right)$$

(14) $$Nu=\frac{HC.{\bar{D}}_{h}}{k_{f}}$$

(15) $$HC=\frac{Q}{A\left(T_{w}-T_{f}\right)}$$

(16) $$R_{t}=\frac{T_{\max}-T_{\min}}{Q}$$

To analyze the overall efficiency of the MCHSs, two different factors of *Nu* and *ΔP* must be studied simultaneously. To do this, a parameter called efficiency index (*η*) is defined by Eq. (17) [25]. Additionally, a criterion for evaluating the overall efficiency of two distinct heat sinks is presented through the use of the relative efficiency index (η_rel_). This criterion allows comparison between the performances of two heat sinks.

(17) $$\eta =\frac{Nu}{{\left(\Delta P\right)}^{1/3}}$$

(18) ηrel=NuNub(ΔPΔPb)1/3

### 2.3 Solution methodology

**Fig. 3:**
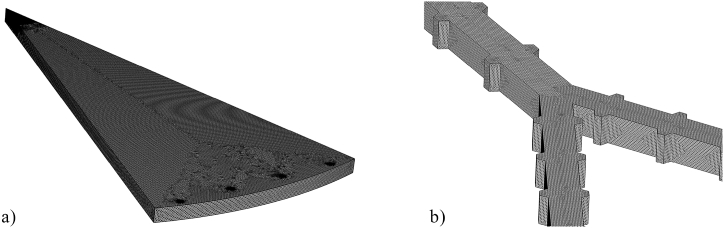
Created mesh on (a) the simulated model and (b) the microchannels and cavities.

**Fig. 4:**
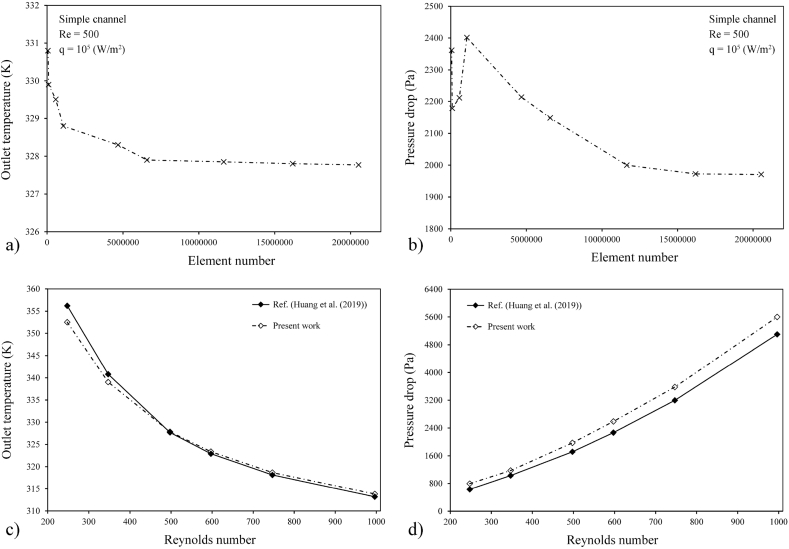
Mesh independency graph for (a) outlet temperature and (b) *ΔP*. And verification data for (c) outlet temperatures and (d) *ΔP*.

COMSOL Multiphysics software was applied to analyze the thermal and hydrodynamic characteristics of the disk-shaped MCHS. Increasing the simulation accuracy in Computational Fluid Dynamics (CFD) analysis involves meshing the domain of interest with smaller elements, which allows for solving the governing equations more effectively [44,45]. Here, the computational domain was meshed by free tetrahedral elements. Different numbers of elements were tested to ascertain the dependency of obtained data from the mesh density. Eventually, the mesh independency was obtained by 16190976 elements in the computational domains. The final created mesh on the simulated model for outlet temperature and *ΔP* data are illustrated in Fig. 3a and b. While, mesh independency graphs are exposed in Fig. 4a and b. The validation process was performed with the work carried out by Huang et al. [25]. The outlet temperature and *ΔP* data acquired herein were compared with the results presented in Ref. [25], as displayed in Fig. 4c and d. Based on this figures, the results achieved herein are in proper accord with the data stated by Ref. [25], and the rationality of the current numerical simulation is verified. Then, various trapezoidal cavities were exerted to the sidewalls of the tree-like microchannels to improve heat transfer characteristics of the disk-shaped MCHS. Re and two different aspect ratios of *t/b* and *h/b* are selected as input parameters. The optimized model of trapezoidal cavities and the best applied inlet velocity were proposed utilizing the RSM and ANN methods.

### 2.4 Response surface methodology

Substantially, different parameters impress the heat transfer behavior of MCHSs, and detecting the most effective one can be helpful in the heat transfer improvement process. The RSM is a specialized statistical method to analyze and optimize the available parameters [46]. It can indicate the interactive influences of process variables and how they subsequently affect outcomes [47,48]. After conducting the experiments and collecting the response data, a mathematical model is then developed to describe the relationship between the input factors and the response variable. In this case, a proper polynomial is chosen as the empirical model to fit the acquired outcomes. The model is analyzed to identify the optimal combination of input variables that would yield the desired response. Overall, RSM is a powerful technique that combines experimental design, statistical analysis, and mathematical modeling to optimize processes, systems, or products [49,50]. Second-order polynomials are the most appropriate equation to fit the investigated data and are defined as follow:

(19) $$Y=\gamma_{0}+\sum_{x=1}^{m}\gamma_{x}X_{x}+\sum_{x=1}^{m-1}\sum_{y=x+1}^{m}\gamma_{xy}X_{x}X_{y}+\sum_{x=1}^{m}\gamma_{xx}X_{x}^{2}+\varepsilon$$

where *γ*_*0*_ indicates the fixed coefficient. *γ*_*x*_, *γ*_*xy*_, and *γ*_*xx*_ denote the coefficients of linear, interaction, and quadratic terms, respectively. *m* and *ε* are allocated to the number of input parameters and statistical error. Besides, *X*_*x*_ and *X*_*y*_ exhibit the input parameters, while *Y* is the acquired response. The interpretation of a higher degree of polynomial equations for RSM design is complex, and the possibility of overfitting the model is considerable. In the present research, the central composite design (CCD) was exerted to build a second-order model for anticipating the *Nu* and *ΔP* as the responses in RSM analysis. All codes were written utilizing the free and open-source programming language of Python (version 3.10). The regression calculations and surface plots were carried out using different Python packages (e.g., Pandas, Numpy, Sklearn, Matplotlib, and Statsmodels). The aspect ratios of *t/b* and *h/b*, and Re are the independent variables. Different sets of configurations were studied to examine the thermo-hydrodynamic properties of the disk-shaped MCHS with hexagonal cavities. To do this, PyDoE2 Python package was applied for the design of experiments (DOE) and to generate experimental conditions based on the CCD. The levels and code of variables are listed in Table 3, and the CCD-provided runs are presented in Table 4.

**Table 3:** Parameters and their allowable levels.

Parameter	Coded parameters	Parameter levels		
		−1	0	+1
*t/b*	*A*	0.2	0.5	0.8
*h/b*	*B*	0.2	0.5	0.8
*Re*	*C*	300	650	1000

**Table 4:** The CCD-provided experiments.

Run	Variables		
	t/b	h/b	Re
1	0.8	0.8	1000
2	0.8	0.5	650
3	0.2	0.8	300
4	0.8	0.2	300
5	0.5	0.5	300
6	0.5	0.2	650
7	0.5	0.8	650
8	0.5	0.5	650
9	0.2	0.5	650
10	0.2	0.2	1000
11	0.8	0.8	300
12	0.2	0.8	1000
13	0.2	0.2	300
14	0.8	0.2	1000
15	0.5	0.5	1000

### 2.5 Artificial neural network

**Fig. 5:**
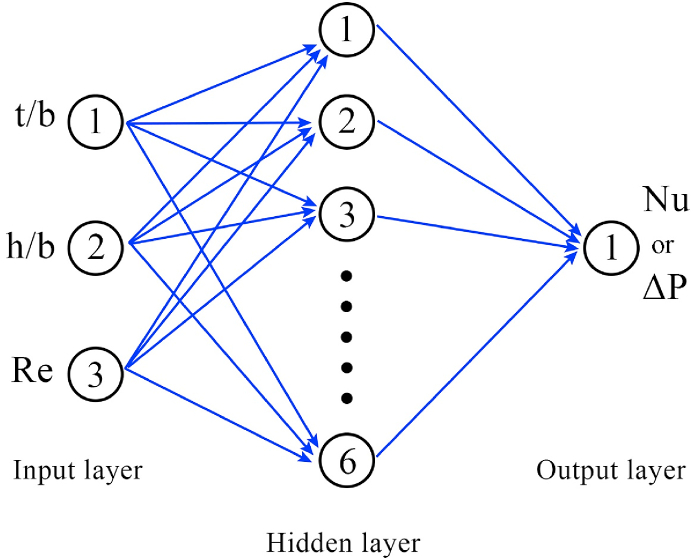
The structure of the applied ANN.

The ANN is employed as a computational method for replicating the actions of neuron-based systems and constructing representations of intricate nonlinear functions [51,52]. This method generally performs controlled learning tasks, building knowledge from data sets where the correct response is presented forward [53,54]. The networks are then trained to detect the correct response, enhancing the precision of their anticipations [55]. Each network includes three specific layers of an input, an output, and some hidden based on the complexity of the process. In the current work, Python software was applied to optimize selected networks' weights and biases. Fig. 5 displays the schematic of the ANN structure of this network. Two distinct 3-6-1 networks are applied for each response (the *Nu* and *ΔP*). In each network, there are a three-neuron input layer, a six-neuron hidden layer, and a one-neuron output layer. To investigate the independency of the predicted results from the neuron numbers, the number of various neurons in the hidden layer of the ANN was analyzed, and it was realized that the neural network with 6 neurons in its hidden layer had the highest accuracy for both responses. Besides, it could be deduced that there was no noticeable change in the network with the increase of neurons numbers. While, with the enhancement of neuron number to more than 6, the possibility of network's overfitting increased. In these networks, the tangent sigmoid and linear transfer functions are regarded for the hidden and output layers, respectively. In both RSM and ANN analysis, the accuracy of the model should be assessed. The coefficient of determination (R^2^), the root mean square error (RMSE), and the normalized standard deviation (Δq %) are statistical measures employed to assess the accuracy of the models offered by ANN and RSM, as follow [56,57]:

(20) $$R^{2}=1-\frac{\sum_{i=1}^{m}{\left(r_{num}-r_{pred}\right)}^{2}}{\sum_{i=1}^{m}{\left(r_{num}-\bar{r_{pred}}\right)}^{2}}$$

(21) $$RMSE=\sqrt{\frac{\sum_{i=1}^{m}{\left(r_{pred}-r_{num}\right)}^{2}}{m}}$$

(22) $$\Delta q=100\times \sqrt{\sum_{i=1}^{m}{\left(1-\frac{r_{pred}}{r_{num}}\right)}^{2}}$$

## 3 Results and discussion

**Fig. 6:**
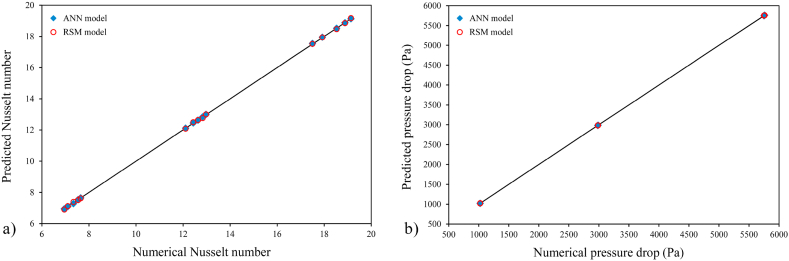
Comparison between the predicted and the actual values of the (a) *Nu* and (b) *ΔP*.

**Fig. 7:**
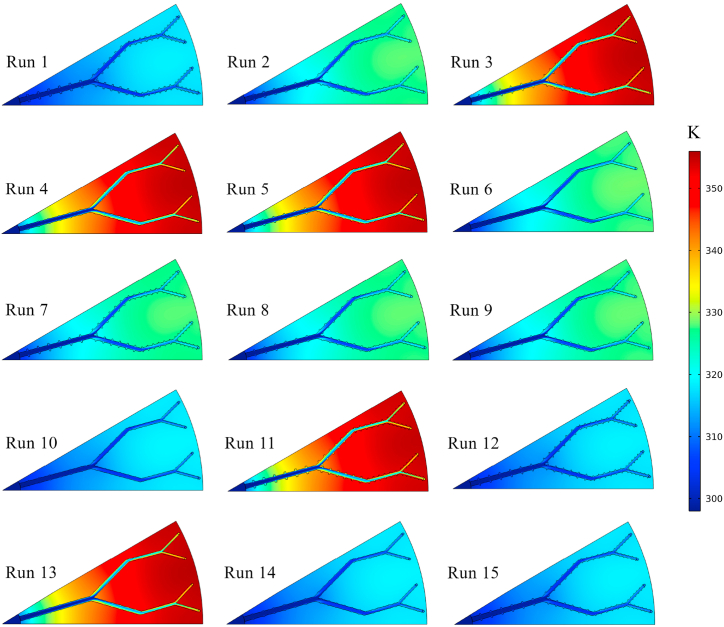
The temperature contours of the MCHS in every 15 runs.

**Fig. 8:**
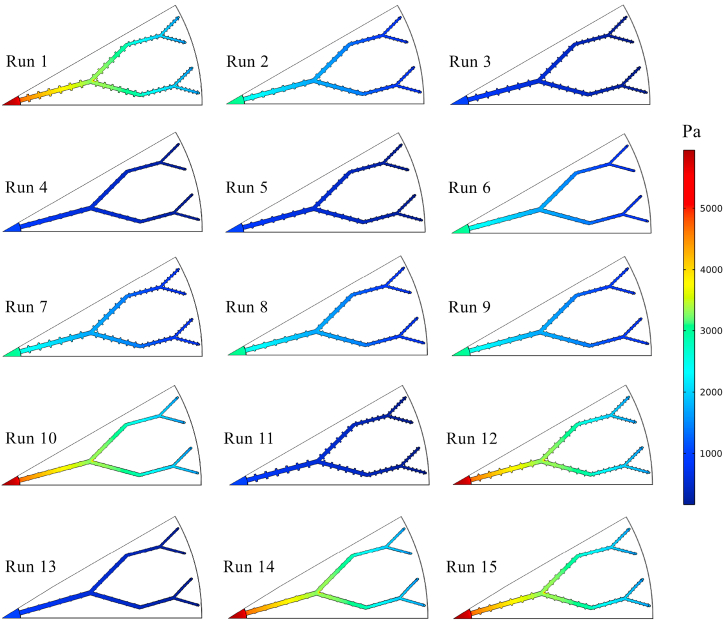
The pressure contours of the MCHS in every 15 runs.

**Fig. 9:**
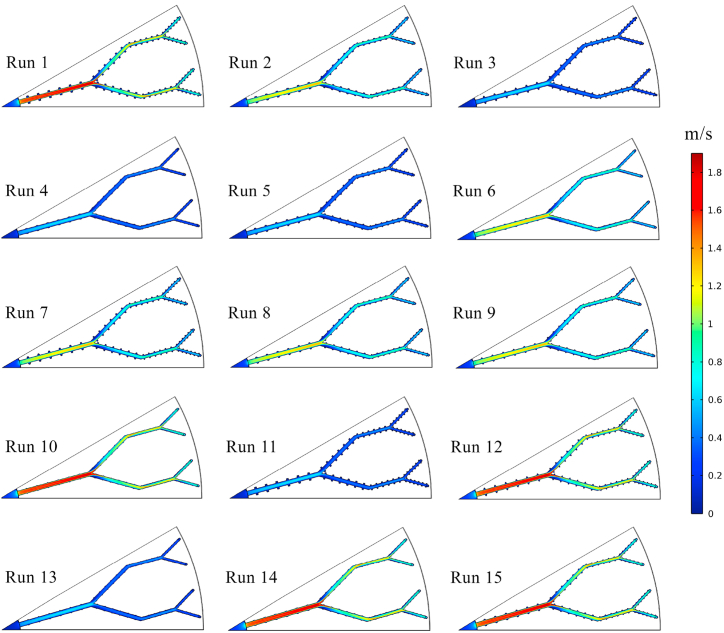
The velocity contours of the MCHS in every 15 runs.

In the present study, the thermo-hydrodynamic performance of the disk-shaped MCHS was evaluated by considering *Nu* and *ΔP* as the response variables. The values of *Nu* and *ΔP* were determined through numerical simulations. Subsequently, the RSM and ANN methods were employed to predict the results of these variables, as shown in Table 5. The data provided indicates that in the majority of simulation runs, the responses obtained from the ANN method exhibit a higher degree of similarity to the results of numerical simulations compared to those obtained from the RSM method. Nevertheless, it is noticeable that both RSM and ANN methods are capable of accurately predicting the responses to a considerable extent. The highest *Nu* value (19.1357) was achieved in the 10th numerical run, specifically at *t/b* = 0.2, *h/b* = 0.2, and Re = 1000. Furthermore, when evaluating the same combination of variables, both ANN and RSM predictions yield the highest *Nu* values. A higher *Nu* value indicates better heat transfer performance of the heat sinks. However, it is crucial to assess the *ΔP* of the device alongside its thermal behavior. Based on the data presented in Table 5, the 10th numerical run demonstrates the highest *ΔP* values across all three models (numerical, ANN, and RSM). Conversely, when the objective of the present investigation is to achieve the lowest *ΔP* in MCHS, the configurations of the 4th numerical run are proposed. Therefore, it is necessary to establish a comprehensive optimization criterion, referred to as the efficiency index in this research, based on both the highest *Nu* and the lowest *ΔP* values. For an easier comparison between the predicted and the actual values of the *Nu* and *ΔP*, Fig. 6a and b are also presented. In order to illustrate the thermo-hydrodynamic characteristics of the disk-shaped MCHS, the temperature, pressure, and velocity distributions in the microchannels for each of the fifteen runs are depicted in Fig. 7, Fig. 8, Fig. 9, respectively. These figures display the distributions at a distance of 0.45 mm from the bottom wall of the heat sink. As evident in Fig. 7, the heat transfer improves as the Reynolds number increases, resulting in a more uniform temperature distribution. With a constant heat flux applied, higher mass flow rates (higher *Re*) lead to a reduction in the temperature difference between the inlet and outlet. Consequently, the cooling process in the MCHS is more effective. The pressure distribution within the tree-like microchannels of the heat sink indicates that the use of trapezoidal cavities does not significantly impact the *ΔP* of the device, as shown in Fig. 8. Additionally, it is apparent that higher values of *ΔP* occur with increasing Reynolds number. The velocity of water within the microchannels is highest at the center and increases as the flow becomes fully developed along the channels. However, when the water passes through the cavities, it gets trapped inside them, resulting in a decrease in velocity, as depicted in Fig. 9.

**Table 5:** The comparison between *Nu* and *ΔP* data calculated from numerical simulation, and their results which have been predicted by ANN, and RSM.

Run	*Nu*			*ΔP* (Pa)		
	Numerical	RSM	ANN	Numerical	RSM	ANN
1	17.4997	17.5187	17.5413	5752.0000	5751.6700	5752.0000
2	12.4304	12.4978	12.4490	2982.9000	2984.0300	2982.9000
3	7.0976	7.1238	7.0982	1023.5000	1022.6800	1023.5000
4	7.5342	7.5139	7.5373	1022.1000	1020.2500	1022.1000
5	7.3411	7.3949	7.2846	1023.5000	1025.6100	1023.5000
6	12.9732	12.9982	12.9952	2982.0000	2984.0000	2982.0000
7	12.1043	12.0788	12.1108	2982.7900	2981.8600	2983.7000
8	12.6356	12.6353	12.6242	2987.5600	2985.4200	2987.6000
9	12.8327	12.7648	12.8401	2985.7000	2985.6500	2986.1000
10	19.1357	19.1759	19.1367	5761.0000	5760.2300	5761.0000
11	6.9523	6.9116	6.9537	1023.6000	1024.1100	1023.6000
12	17.9195	17.9392	17.9539	5750.5000	5752.0900	5750.5000
13	7.6470	7.6274	7.6485	1023.0000	1023.0700	1023.4000
14	18.8807	18.8540	18.8786	5755.0000	5755.5600	5755.0000
15	18.5270	18.4727	18.5226	5759.0000	5757.9600	5757.7000

As mentioned earlier, quadratic polynomials are considered the most suitable equations for constructing a reliable model. The quadratic polynomial equations for the two responses, *Nu* and *ΔP*, take the form of Eqs. (23), (24), correspondingly. These equations indicate the normalized state of the input variables. These equations, along with the ANOVA tables for analyzing the results, have been generated using Python. The ANOVA tests were employed to assess the accuracy of the regression models. The outcomes of the ANOVA tests for the quadratic models of *Nu* and *ΔP* in the normalized state of the input variables can be found in Table 6, Table 7, respectively. The P-value obtained from the ANOVA tests serves as a criterion to determine that the test results are statistically significant. A lower P-value (below 0.05) for a specific ratio indicates a higher level of confidence in stating that a parameter is not significant. Table 6, Table 7 provide an overview of the significance of each variable (*t/b*, *h/b*, and *Re*) as well as the interactions between them. The actual state of the mentioned equations is also presented by Eqs. (25), (26).

(23) $$Nu=12.6355-0.1335\times A-0.4597\times B+5.5390\times C-0.0247\times A\times B-0.0521\times A\times C-0.1832\times B\times C-0.0040\times A^{2}-0.0968\times B^{2}+0.2985\times C^{2}$$

(24) $$\Delta P=2985.4220-0.8100\times A-1.0710\times B+2366.1800\times C+1.0625\times A\times B-0.4625\times A\times C-1.9375\times B\times C-0.5878\times A^{2}-2.4928\times B^{2}+406.3622\times C^{2}$$

**Table 6:** ANOVA table for *Nu* model.

Dep. Variable: Nusselt number			*R*^*2*^: 0.999		
Model: Ordinary least squares			F-statistic: 7.651x10^3^		
Method: Least Squares			Prob (F-statistic): 8.37x10^−10^		
Sources	coefficient	standard error		T-value	P-value
const	12.6355	0.0360		350.5370	0.0000
*A (t/b)*	−0.1335	0.0210		−6.2960	0.0010
*B (h/b)*	−0.4597	0.0210		−21.6780	0.0000
*C (Re)*	5.5390	0.0210		261.1800	0.0000
*AB*	−0.0247	0.0240		−1.0400	0.3460
*AC*	−0.0521	0.0240		−2.1970	0.0790
*BC*	−0.1832	0.0240		−7.7280	0.0010
*A*^*2*^	−0.0040	0.0420		−0.0950	0.9280
*B*^*2*^	−0.0968	0.0420		−2.3140	0.0690
*C*^*2*^	0.2985	0.0420		7.1380	0.0010

**Table 7:** ANOVA table for *ΔP* model.(25)Nu=3.29033+5.8553×10−2×(tb)+0.81425×(hb)+1.3778×10−2×Re−0.27412×(tb)×(hb)−4.95973×10−4×(tb)×Re−1.74524×10−3×(hb)×Re−4.4149×10−2×(tb)2−1.07522×(hb)2+2.43695×10−6×Re2(26)ΔP=−17.27633+0.79118×(tb)+30.2188×(hb)+2.45953×Re+11.80556×(tb)×(hb)−4.40476×10−3×(tb)×Re−1.8452×10−2×(hb)×Re−6.53086×(tb)2−27.69753×(hb)2+3.31724×10−3×Re2

Dep. Variable: Pressure drop			*R*^*2*^: 0.999		
Model: Ordinary least squares			F-statistic: 1.303x106		
Method: Least squares			Prob (F-statistic): 2.21x10^−15^		
Sources	coefficient	standard error		T-value	P-value
const	2985.4220	1.1800		2529.2790	0.0000
*A (t/b)*	−0.8100	0.6940		−1.1660	0.2900
*B (h/b)*	−1.0710	0.6940		−1.5420	0.1840
*C (Re)*	2366.1800	0.6940		3407.2510	0.0000
*AB*	1.0625	0.7760		1.3680	0.2290
*AC*	−0.4625	0.7760		−0.5960	0.5770
*BC*	−1.9375	0.7760		−2.4950	0.0550
*A*^*2*^	−0.5878	1.3690		−0.4290	0.6860
*B*^*2*^	−2.4928	1.3690		−1.8200	0.1280
*C*^*2*^	406.3622	1.3690		296.7270	0.0000

**Fig. 10:**
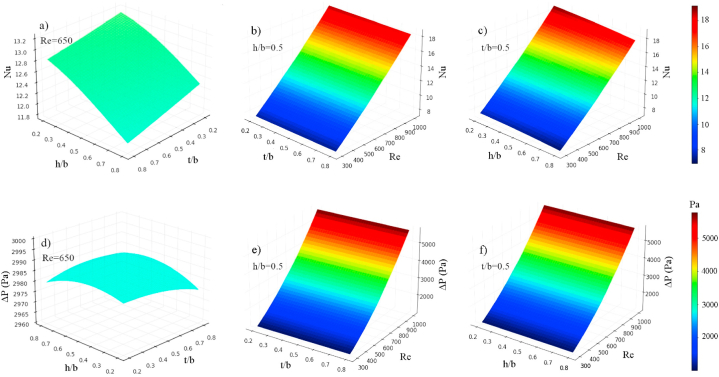
The 3D contours of *Nu* and *ΔP*.

**Fig. 11:**
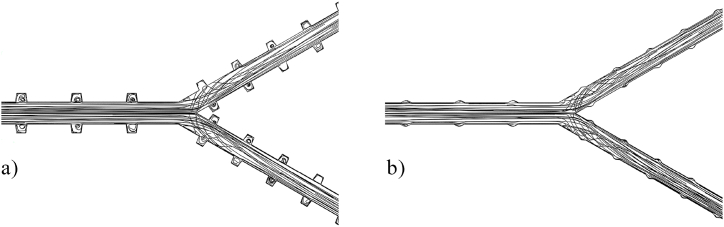
Streamlines of the microchannel together with its trapezoidal cavities for (a) the 1st and (b) the 10th configurations.

Fig. 10 presents the 3D contours illustrating the precise influence of each variable on the *Nu* and *ΔP*. These three-dimensional surface plots were generated using Python packages. The contours visually depict the combined effects of the aspect ratios of *t/b* and *h/b*, as well as the Reynolds number, on both *Nu* and *ΔP*. Based on the analysis of the figures, it can be observed that the aspect ratios of *t/b* and *h/b* have a relatively minor impact on the *Nu* and *ΔP* of the device compared to the Reynolds number. This suggests that modifying the sides or height of the hexagonal cavities does not significantly affect the heat transfer and pressure drop of the MCHS. Indeed, as previously demonstrated by the temperature and pressure contours in Fig. 7, Fig. 8, it is apparent that the Reynolds number is the most influential variable affecting both the *Nu* and *ΔP*. Increasing the Reynolds number leads to a significant enhancement in heat transfer and pressure drop in the device. Taking into account the minimal impact of the aspect ratios *t/b* and *h/b* on the *Nu*, as depicted in Fig. 10a, it can be observed that increasing the upper side or height of the trapezoidal cavities leads to a decrease in *Nu*. Despite the fact that enlarging the cavity area enhances heat transfer in the heat sink, it is noted that the heat transfer coefficient and, consequently, *Nu* decrease in larger cavities. This suggests that there is a trade-off between cavity size and heat transfer performance, where larger cavities may not necessarily result in improved heat transfer coefficients. Fig. 10b and c demonstrate that the *Nu* is more responsive to changes in the Reynolds number compared to the aspect ratios of *t/b* and *h/b*. Increasing Re leads to an enhancement in *Nu*. To further explore the impact of altering the size of the upper side (*t/b*) or the height (*h/b*) of the trapezoidal cavities on the *ΔP*, Fig. 10d is provided. It is evident that increasing the aspect ratio of *t/b* results in a decrease in *ΔP*, indicating that larger values of *t/b* contribute to reduced pressure drops in the system. Interestingly, two different trends are observed when varying the aspect ratio of *h/b*. Initially, the *ΔP* increases as the aspect ratio of *h/b* rises (from 0.2 to 0.5). However, beyond a certain point, the relationship between *ΔP* and the aspect ratio of *h/b* becomes inverse, indicating that further increases in *h/b* result in decreased pressure drops. However, it should also be noted that the effect of these geometrical parameters on pressure drop is low compared to *Re*. The direct relationship between the Reynolds number and *ΔP* is demonstrated in Fig. 10e and f, confirming that higher Re values are associated with higher pressure drops. Furthermore, Fig. 11 presents the streamlines of the tree-like microchannel and its trapezoidal cavities with the settings of the 1st and 10th Run. When considering the 1st settings (*t/b* = 0.8, *h/b* = 0.8, and Re = 1000), the trapezoidal cavities are larger, causing the water to flow into the cavities and become momentarily trapped, as depicted in Fig. 11a. This generates a recirculation flow that improves heat transfer from the cavity walls to the water, thereby enhancing the cooling capacity of the device. Nevertheless, according to the illustration shown in Fig. 11b, the surface area of the cavities is smaller (with *t/b* = 0.2, *h/b* = 0.2, and Re = 1000) and it does not alter the direction of water flow. Consequently, this results in a slight reduction in thermal performance during the 10th run compared to the initial configuration in the 1st run. It can be concluded that the *Nu* value in the 10th run is higher due to the heat transfer being unable to adequately compensate for the increase in the heat transfer surface.

As outlined in section 2.5, the ANN methodology was employed to predict the *Nu* and *ΔP* in a disk-shaped MCHS. For each response, a separate network with a topology of 3-6-1 was utilized. The selection of the transfer function in the network is crucial for accurately capturing the relationship between the inputs and outputs of the system. In this study, the tangent sigmoid transfer function was chosen for the hidden layer of the networks. Due to the limited ranges of [−1, +1] for the tangent sigmoid transfer function and [0, +1] for the log-sigmoid transfer function, they were not suitable for the output layer. To address this limitation, alternative transfer functions were explored and the linear transfer function was selected for the output layer. Various weights and biases were tested to minimize the error between the data sets and the model output, ensuring a more accurate prediction. Table 8 presents the suitable weights and biases for the optimal training of the ANN model in predicting the *Nu*. The corresponding equations for the weights and biases can be found in Eqs. (27), (28). Similarly, Table 9 displays the derived values for the ANN model in predicting the *ΔP*, along with the associated Eqs. (29), (30). These tables provide valuable information for the implementation and performance evaluation of the ANN models for both *Nu* and *ΔP*.

**Table 8:** Optimized weights and biases for the ANN model of *Nu*.(27)$$Nu=\left(\left[0.0169-0.0237-0.1226\;0.1491\;0.6186-0.7911\right]\times {\left[\begin{array}{c} \frac{2}{1+\exp\left(-2P_{1}\right)}-1\\ \vdots \\ \frac{2}{1+\exp\left(-2P_{6}\right)}-1 \end{array}\right]}_{Nu}+\left[-0.0668\right]+1\right)\times \frac{19.1357-6.9523}{2}+6.9523$$(28)$${\left[\begin{array}{c} P_{1}\\ P_{2}\\ P_{3}\\ P_{4}\\ P_{5}\\ P_{6} \end{array}\right]}_{Nu}=\left[\begin{array}{ccc} 0.6858 & -0.2466 & -0.0362\\ -0.4676 & 0.8261 & -0.0314\\ 0.1823 & 0.6547 & 0.1511\\ -0.0694 & -0.4856 & 0.4268\\ 0.0169 & 0.1106 & 0.6302\\ 0.0378 & 0.0156 & -0.8724 \end{array}\right]\times \left[\begin{array}{c} 2\times \frac{\frac{t}{b}-0.2}{0.8-0.2}-1\\ 2\times \frac{\frac{h}{b}-0.2}{0.8-0.2}-1\\ 2\times \frac{Re-300}{1000-300}-1 \end{array}\right]+\left[\begin{array}{c} -1.1629\\ -1.0116\\ -0.7047\\ -0.282\\ 0.2332\\ 0.2313 \end{array}\right]$$

Hidden layer				Output	
Weights			Biases	Weights	Bias
0.6858	−0.2466	−0.0362	−1.1629	0.0169	−0.0668
−0.4676	0.8261	−0.0314	−1.0116	−0.0237	
0.1823	0.6547	0.1511	−0.7047	−0.1226	
−0.0694	−0.4856	0.4268	−0.2820	0.1491	
0.0169	0.1106	0.6302	0.2332	0.6186	
0.0378	0.0156	−0.8724	0.2313	−0.7911

**Table 9:** Optimized weights and biases for the ANN model of *ΔP*.(29)$$\Delta P=\left(\left[-0.0044-0.7783-0.0018\;0.0569-0.6375\;0.0779\right]\times {\left[\begin{array}{c} \frac{2}{1+\exp\left(-2P_{1}\right)}-1\\ \vdots \\ \frac{2}{1+\exp\left(-2P_{6}\right)}-1 \end{array}\right]}_{\Delta P}+\left[0.0643\right]+1\right)\times \frac{5761-1022.1}{2}+1022.1$$(30)$${\left[\begin{array}{c} P_{1}\\ P_{2}\\ P_{3}\\ P_{4}\\ P_{5}\\ P_{6} \end{array}\right]}_{\Delta P}=\left[\begin{array}{ccc} 0.6294 & 0.4606 & 0.7078\\ -0.0051 & -0.0054 & -1.0853\\ -0.4636 & -0.3333 & -0.9261\\ 0.1435 & -0.1931 & 0.5175\\ 0.0082 & 0.0067 & -0.6800\\ -0.0616 & 0.1750 & 0.7362 \end{array}\right]\times \left[\begin{array}{c} 2\times \frac{\frac{t}{b}-0.2}{0.8-0.2}-1\\ 2\times \frac{\frac{h}{b}-0.2}{0.8-0.2}-1\\ 2\times \frac{Re-300}{1000-300}-1 \end{array}\right]+\left[\begin{array}{c} -0.8516\\ 0.4872\\ -0.3298\\ 0.6342\\ -0.0586\\ 0.6402 \end{array}\right]$$

Hidden layer				Output	
Weights			Biases	Weights	Bias
0.6294	0.4606	0.7078	−0.8516	−0.0044	0.0643
−0.0051	−0.0054	−1.0853	0.4872	−0.7783	
−0.4636	−0.3333	−0.9261	−0.3298	−0.0018	
0.1435	−0.1931	0.5175	0.6342	0.0569	
0.0082	0.0067	−0.6800	−0.0586	−0.6375	
−0.0616	0.1750	0.7362	0.6402	0.0779	

The RSM and ANN models were individually employed to identify the optimal heat transfer and *ΔP* in a disk-shaped MCHS with trapezoidal cavities. To assess the validity and accuracy of these models, specific statistical factors defined in Eqs. (20), (21), (22) were utilized, and the corresponding results are presented in Table 10. The coefficient of determination quantifies the extent to which the variation in the responses can be explained by the input parameters. As the coefficient of determination value approaches 1, it indicates a strong correlation between the input and output variables, suggesting a satisfactory relationship between them. Both the RSM and ANN models exhibited excellent performance in predicting the *Nu* and *ΔP* of the MCHS, with a coefficient of determination of 0.999 for each response. This high coefficient of determination indicates that both models accurately captured 99.9 % of the variations in *Nu* and *ΔP*. Table 11 presents the optimal configurations of trapezoidal cavities and the recommended Reynolds number for achieving the highest *Nu* or the lowest *ΔP* using the RSM and ANN models. The table provides details of the configurations associated with the maximum *Nu* or minimum *ΔP* for both the RSM and ANN methods. To achieve the maximum efficiency index, which entails optimal heat transfer and the lowest *ΔP* simultaneously, both the RSM and ANN models recommended utilizing the aspect ratios of *t/b* = 0.2, *h/b* = 0.2, and a Reynolds number of 1000. With these fixed values for the independent variables in both models, the efficiency index was obtained as approximately 1.07 and 1.067 using the RSM and ANN approaches, respectively. The close proximity of the optimal values obtained from both the ANN and RSM methods highlights their high accuracy in predicting the best-performing model. Furthermore, it is observed that the MCHS with the aspect ratios of *t/b* = 0.8, *h/b* = 0.8, and a Reynolds number of 300 exhibits the weakest overall performance, as indicated by the minimum efficiency index, according to the models derived from the ANN and RSM methods. The relative efficiency index parameter is utilized to quantify the difference between the maximum and minimum efficiency index. According to the predictions of the ANN and RSM models, considering the relative efficiency index, the performance of the MCHS in its most optimal state is projected to be 54.6 % and 56 % superior to the performance of the device in its worst state, respectively.

**Table 10:** The Comparison between the RSM and ANN models using statistical parameters.

Error function	*Nu*		*ΔP*	
	RSM	ANN	RSM	ANN
*R*^*2*^	0.999	0.999	0.999	0.999
RMSE	0.039	9.47 × 10^−5^	1.268	0.593
*Δq* (%)	1.409	0.004	0.312	0.201

**Table 11:** Optimized values of input parameters for each response.

Optimized parameter	RSM				ANN			
	Value	*t/b*	*h/b*	*Re*	Value	*t/b*	*h/b*	*Re*
*Nu* (maximum)	19.176	0.2	0.2	1000	19.135	0.2	0.2	1000
*ΔP* (minimum)	1020.2	0.8	0.2	300	1021.6	0.8	0.2	300
*η* (maximum)	1.07	0.2	0.2	1000	1.067	0.2	0.2	1000

After finding the optimum state for the copper-made MCHS (containing the optimum design of the cavities along with the best applied velocity), different materials were tested and compared with the base case (heat sink made of copper). Table 12 presents the obtained data for the various MCHSs made of different materials. According to this table, the pressure drop in the mentioned heat sinks is not significantly different. Because the overall geometry and the applied velocity are the same in all of these devices, and the only difference is in the fluid viscosity (due to the slight changes in temperature), the pressure drop remains nearly constant. Considering the thermal behavior, it is evident that as the thermal conductivity decreases from copper to alumina, the Nusselt number decreases and leads to an increase in the total thermal resistance of heat sinks. Thermal resistance is an important measure of the performance of the MCHSs, and its increment indicates a decrease in the cooling power of these devices. Based on the relative efficiency index parameter (*η*_*rel*_*)* discussed in this section, which serves to compare the performance of heat sinks made of various materials with that of a copper heat sink, it is evident that all heat sinks exhibited values below 1. This indicates a decrease in overall performance relative to the copper heat sink. Notably, the Alumina heat sink demonstrated the highest performance loss, with *η*_*rel*_ value of 0.8993, indicating a decrease in overall performance by 10.07 % compared to the copper heat sink. It is essential to acknowledge that ceramic materials (TiB_2_, ZrB_2_, and Alumina) possess distinct properties. These materials exhibit significantly higher melting points and are corrosion-resistant, making them suitable for use in challenging conditions with corrosive fluids [1].

**Table 12:** A comparison between the thermo-hydraulic properties of heat sinks made of different materials.

Material of optimal design	*Nu*	*R*_*t*_ (K/W)	*ΔP* (Pa)	*η*	*η*_*rel*_
Copper	19.1357	1.0612	5761.0000	1.0674	1
Aluminum	18.7561	1.1678	5761.5000	1.0462	0.9801
Silicon	18.5184	1.2370	5761.9000	1.0330	0.9677
TiB_2_	18.1627	1.3748	5762.3000	1.0130	0.9491
ZrB_2_	18.0405	1.4213	5762.4000	1.0063	09427
Alumina	17.2098	1.9572	5762.8000	0.9599	0.8993

## 4 Conclusions

The investigated geometry chosen for numerical analysis was a bionic fractal MCHS. To enhance the heat transfer properties of the device, several trapezoidal cavities were incorporated into the microchannels. The analysis of the investigated geometry and the solution of the equations were performed using the COMSOL Multiphysics Software. In order to predict the optimal cooling capacity of the disk-shaped MCHS, the ANN and RSM techniques were employed. The thermal and flow properties of the device were modeled separately using properly designed versions of the ANN and RSM methods. All the codes were written using Python. The independent variables considered in the analysis were the aspect ratios of *t/b* and *h/b*, as well as the Reynolds number. On the other hand, the responses examined in this study were the *Nu* and the *ΔP*. After finding the optimum state for the copper-made MCHS, different materials were tested and compared with the base case (heat sink made of copper). Based on the obtained results.

• The 3D contours of the *Nu* indicated that the aspect ratios of *t/b* and *h/b* had a negligible influence on *Nu* compared to the Reynolds number.
• Increasing the upper side or height of the trapezoidal cavities led to a decrease in *Nu.*
• The results obtained from the ANN exhibited a closer agreement with the numerical model outcomes compared to those derived from the RSM. Nevertheless, it is important to emphasize that both the ANN and RSM models accurately predicted the thermal and flow properties of the MCHS.
• The coefficient of determination (*R*^*2*^), which had a value of 0.999, indicated that both the RSM and ANN models accurately captured 99.9 % of the variations in the responses.
• When considering the aspect ratios of *t/b* = 0.2, *h/b* = 0.2, and a Reynolds number of 1000, the optimal efficiency index (which encompasses higher heat transfer and lower *ΔP* simultaneously) was achieved at approximately 1.07 and 1.067 using the RSM and ANN models, respectively.
• Through the utilization of the relative efficiency index and predictions from the ANN models, it was determined that the performance of the most optimal design exhibited a 54.6 % improvement when compared to the design with the weakest performance. Similarly, the models generated from the RSM method predicted this improvement to be approximately 56 %.
• Among the different investigated materials, with the decrease in thermal conductivity, the Nusselt number decreases and led to an increase in the total thermal resistance of heat sinks. The increment in thermal resistance indicated a decrease in the cooling power of these devices. The Alumina heat sink has the weakest performance, with a 10.07 % decrease in overall performance compared to the copper heat sink.

## Data availability statement

Data will be made available on request.

## Additional information

No additional information is available for this paper.

## CRediT authorship contribution statement

**Kourosh Vaferi:** Writing – review & editing, Writing – original draft, Investigation. **Mohammad Vajdi:** Writing – review & editing, Writing – original draft, Supervision, Project administration. **Sahar Nekahi:** Writing – review & editing, Writing – original draft, Investigation, Conceptualization. **Amir Heydari:** Writing – review & editing, Writing – original draft, Formal analysis, Conceptualization. **Farhad Sadegh Moghanlou:** Writing – review & editing, Writing – original draft, Supervision, Project administration. **Hossein Nami:** Writing – review & editing, Writing – original draft, Supervision, Project administration, Funding acquisition. **Haleh Jafarzadeh:** Writing – review & editing, Writing – original draft, Supervision.

## Declaration of competing interest

The authors declare that they have no known competing financial interests or personal relationships that could have appeared to influence the work reported in this paper.

## Nomenclature

*A*: heat transfer area (m^2^)
*b*: length of cavities bottom side (m)
*C*_*p*_: heat capacity (J/kg.K)
*D*_*h*_: hydraulic diameter (m)
*H*_*c*_: height of microchannels (m)
*H*_*h*_: height of heat sink (m)
h: height of cavities (m)
*HC*: heat transfer coefficient (W/m^2^.K)
*k*: thermal conductivity (W/m.K)
*L*: length of microchannels (m)
*m*: number of experiments
*ṁ*: mass flow rate (kg/s)
*Nu*: Nusselt number
*ΔP*: pressure drop (Pa)
*Q*: transferred heat (W)
*R*_*h*_: radius of the heat sink (m)
*R*_*i*_: radius of the heat sink's inlet (m)
*R*_*o*_: radius of the heat sink's outlet (m)
*R*_*t*_: total thermal resistance (K/W)
*Re*: Reynolds number
r: response
*S*_*ci*_: distance between cavities (m)
*S*_*s0*_: distance between inlet and first cavity (m)
*T*: temperature (K)
*t*: length of cavities upper side (m)
*U*: velocity (m/s)
*W*: width of microchannels (m)
*θ*: expansion angle of microchannels (degree)
*α*: closed angles inside cavities (degree)
*β*: constant geometrical coefficient
*β′*: constant geometrical coefficient
*β’’*: constant geometrical coefficient
*ρ*: density (kg/m^3^)
*μ*: dynamic viscosity (Pa.s)
*η*: efficiency index

**Subscripts**
*b*: base
*c*: microchannel
*f*: fluid
*h*: heat sink
*i*: microchannel level number
*in*: inlet
*num*: numerical
*out*: outlet
*pred*: predicted
*rel*: relative
*s*: solid
*w*: wall

**Abbreviations**
ANN: artificial neural network
ANOVA: analysis of variance
CFD: computational fluid dynamic
DOE: design of experiments
MCHS: microchannel heat sink
RMSE: root mean squared error
RSM: response surface methodology
